# Enzymatic Desymmetrisation of Prochiral *meso*-1,2-Disubstituted-1,2-Diaminoethane for the Synthesis of Key Enantioenriched (−)-Nutlin-3 Precursor

**DOI:** 10.3390/molecules29143267

**Published:** 2024-07-10

**Authors:** Virginia Cristofori, Davide Illuminati, Chiara Bisquoli, Martina Catani, Greta Compagnin, Giulia Turrin, Claudio Trapella, Anna Fantinati

**Affiliations:** 1 Department of Chemical, Pharmaceutical and Agricultural Sciences, University of Ferrara, Via Luigi Borsari, 46, 44121 Ferrara, Italy; virginia.cristofori@unife.it (V.C.); chiara.bisquoli@unife.it (C.B.); martina.catani@unife.it (M.C.); greta.compagnin@unife.it (G.C.); 2 Department of Life Sciences, University of Modena and Reggio Emilia, Via G. Campi 213/d, 41125 Modena, Italy; davide.illuminati@unife.it; 3 Department of Environmental and Prevention Sciences, University of Ferrara, Corso Ercole I d’Este, 32, 44121 Ferrara, Italy; giulia.turrin@unife.it (G.T.); anna.fantinati@unife.it (A.F.)

**Keywords:** biocatalysis, Nutlin-3a, desymmetrisation, vicinal *meso*-diamines

## Abstract

Herein we present the biocatalysed preparation of a mono-*N*-carbamate-protected precursor of antitumoral Nutlin-3a through enantioselective alkoxycarbonylation of *meso*-1,2-disubstituted-1,2-diaminoethane using enzyme lipases and dialkyl carbonates as acylating agents. A series of supported or free lipase enzymes were screened in combination with commercially available diallyl, diethyl and dimethyl carbonates. The reactions were conducted at different temperatures, for different reaction times and with variable co-solvent systems to evaluate the effects on the enzyme catalytic activity. The best results in terms of conversion, enantiomeric excess and yield were obtained when lipase from *Candida antarctica B* (CAL-B) was used with diallyl carbonate (DAC) when conducting the reaction solventless at 75 °C.

## 1. Introduction

Thanks to its role in cell cycle stabilisation and DNA integrity, the protein p53 is often called “the genome guardian”. Its function as a tumour suppressor is widely recognised and has been the subject of intense research as a strategic target in cancer therapy [1]. Sophisticated animal models showed that targeting p53 level regulation in order to activate its protective response can be curative, even in advanced tumours [2,3,4]. Among different approaches aimed at the retention and restoration of wild-type p53 function, the blockage of its antagonist oncoprotein MDM2 with small molecule inhibitors as chemotherapeutics is extensively investigated [5,6]. In fact, as the most frequent genomic alteration in cancer, the over-expression or genetic amplification of MDM2 leads to the inactivation of p53 through an auto-regulatory negative feedback loop [7,8].

**Figure 1:**
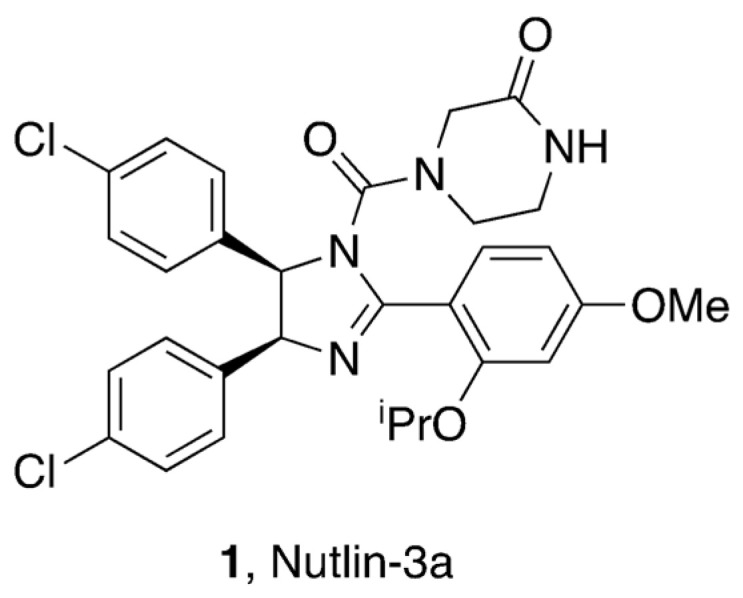
Chemical structure of antitumoral compound Nutlin-3a.

1,2,4,5-tetrasubstituted-4,5-*cis*-imidazolines, also known as Nutlins, are potent and specific inhibitors of the p53-MDM2 protein–protein interaction (PPI) with anti-proliferative activity that inhibit tumour growth. The Nutlins family was developed via high-throughput screening at Hoffmann-La Roche, and the preclinical drug Nutlin-3a, **1** (Figure 1) is one of the most studied compounds [9]. Compound **1** was shown to bind MDM2 right in the p53 pocket with high affinity and represents an appropriate leading compound for ligand structure optimisation [10,11].

Countless studies regarding the use of these heterocyclic drugs in basic cancer biology and clinical trials are nowadays pervasive in the oncology literature [12,13,14,15]. However, their preparation at a large scale is not trivial and requires considerable organic chemistry knacks and suitable facilities, which make them not readily accessible for testing. Furthermore, a quite high eudismic ratio between the levorotatory eutomer (−)-(4S,5R)-Nutlin (also referred to as Nutlin-3a) and the distomer (+)-(4R,5S)-Nutlin (also referred to as Nutlin-3b) demands the enantiomers to be resolved to minimise possible side effects upon the administration of the racemate [16,17].

The original total synthesis of Nutlins initially reported by Hoffmann-La Roche consisted in the preparation of the racemic mixture from benzil (1,2-diphenylethane-1,2-dione) in eight steps and required two time-consuming and expensive chiral supercritical fluid chromatography (SFC) separations for the isolation of the more potent enantiomer [18,19].

**Figure 2:**
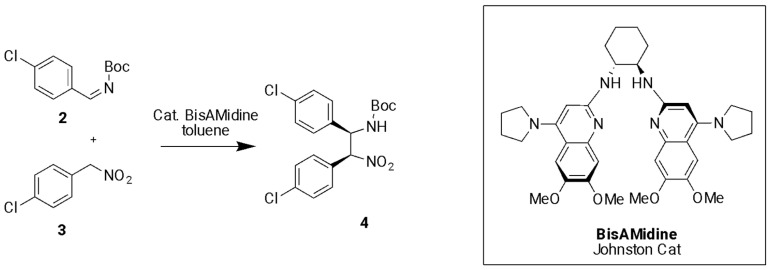
Asymmetric aza-Henry reported by Johnston and co-workers for the synthesis of enantiopure Nutlin-3a.

In 2011, Johnston and co-workers reported the first asymmetric synthesis of **1** through the formation of enantiopure key nitro-amine precursor **4**. The latter was obtained in diastero- and enantioselective fashion via the aza-Henry (also referred to as nitro-Mannich) reaction catalysed by the chiral organocatalyst bisAMidine (Figure 2) [20,21]. Although a high enantiomeric excess of **4** was reached, this synthetic pathway still presents drawbacks and problematic steps in terms of length, costs and efficiency. For instance, the starting materials of the asymmetric step, the aryl *N*-Boc imine **2** and nitromethane **3**, are not easy to prepare, as evidenced by the rather low yields and long reaction time, in addition to the hazardous handling of some needed reactants. Moreover, the scaling up of subsequent chiral nitro compound reduction with the CoCl_2_/NaBH_4_ system seems to have reproducibility issues.

**Figure 3:**
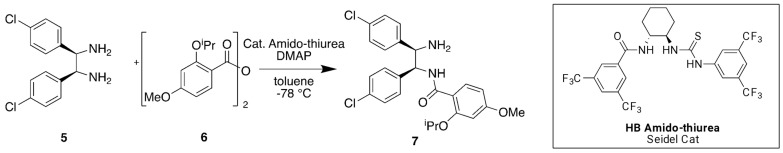
Asymmetric *meso*-diamine desymmetrisation with Seidel HB catalyst implemented by Trapella and co-workers for the synthesis of enantiopure Nutlin-3a.

Later, we reported an alternative expeditious synthesis that allowed us to reduce the number of steps, thus simplifying the overall production process [22]. Our strategy was based on a dual catalytic enantioselective process introduced by Seidel, et al. [23] for the desymmetrisation of prochiral **5**, which is a vicinal *meso*-diamine that, in turn, could be easily obtained in two steps. In particular, the nucleophilic catalyst 4-(dimethylamino)pyridine (DMAP) is used in combination with a chiral hydrogen-bonding (HB) amido-thiourea (Figure 3) [23]. This catalytic system with an aryl anhydride **6** generates in situ an enantiomerically pure tight ion pair, which induces the selectivity for the nucleophilic attack by the *meso*-diamine **5**. A scalemic mixture with an 84:16 ratio of monoamide precursor **7** was achieved using a stoichiometric amount of chiral auxiliary at −78 °C. Although there has been progress in terms of practical optimisation and process simplification of reported strategies, we were keen to further improve the selectivity and yields of the synthesis of (−)-Nutlin-3a **1** and its derivatives.

However, access to enantioenriched or enantiopure monoprotected *cis*-derivatives from *meso*-compound **5**, as the key intermediate for (−)-Nutlin-3a preparation, was proved to be challenging and requires expensive catalysts and time-consuming purifications, besides the environmental issues related to chemical catalysis.

Recent advances in organic synthesis techniques showed that the replacement of chemical approaches with biocatalytic processes is an attractive alternative, as this kind of technology is advantageous in terms of its environmental impact through embracing the green chemistry principles. Enzymes are biodegradable, biocompatible, non-toxic catalysts that allow for operating in less-hazardous settings in comparison with conventional chemical methods. For this reason, enzymes have also been employed in industry in the last 30 years for a wide variety of reactions, often improving the tedious asymmetric syntheses of complex molecules. Due to enzyme efficiency and regio-, enantio- and chemoselectivity, their application is widespread in biocatalysed processes for the preparation of pharmaceuticals or natural bioactive compounds [24].

**Figure 4:**
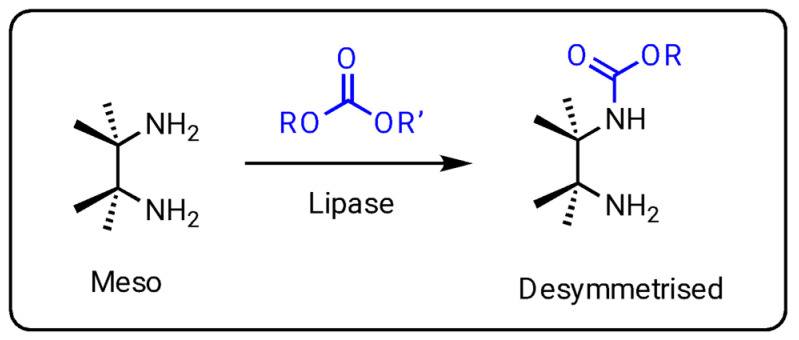
Asymmetric desymmetrisation of prochiral *meso*-diamine biocatalysed by enzyme lipase.

Meaningful exploitations of enzymes revolve around biocatalysed asymmetric processes for the synthesis of optically pure compounds. Among these biotransformations, the enantioselective desymmetrisation of prochiral symmetrical substrates represents an expanding field in biocatalysis [25,26]. While most asymmetric enzymatic reactions proceed through kinetic resolution that can at most reach 50% yield, desymmetrisation carries the advantage that 100% of the substrate can potentially be converted into an enantiopure product. Recently, some enzymatic desymmetrisations, even in continuous-flow mode, have been scaled up for multigram preparative purposes, with several substrates, such as *meso*-diols, ketones or esters, reviewed in the literature [25,26]. However, only a handful of reports describe the enzymatic desymmetrisation of prochiral *meso*-diamines as starting materials [27,28,29,30,31,32]. Even fewer published articles describe vicinal *meso*-diamine as an enzymatic substrate [31,32]. Almost the entirety of these works used lipases, in combination with dialkyl carbonate, to give desymmetrised monocarbamate with high enantiopurity (Figure 4). Lipases are versatile biological catalysts known to display promiscuous activity because of the flexibility of their active site that can accommodate a variety of substrates that are often hydrophobic compounds and structurally diverse in size and shape. The mechanism of the lipase-catalysed formation of carbamate, starting from an amine and a carbonate as substrates, likely involves the typical catalytic triad (serine, histidine, and aspartate or glutamate) present in the lipase active site and proceeds through the formation of an acyl-enzyme intermediate [33]. The enantiopreference of lipase in this case is probably due to the chiral environment surrounding the acyl intermediate that favours the access of nucleophilic diamine in a way that leads to the most snugly pose. As a result, one less-hindered amine will bind effectively, ending in a preferential attack to the acyl donor.

Gotor et al. reported the first stereoselective mono-protection of 2-substituted 1,3-propanediamine derivatives through a *Pseudomonas cepacia* (PSL-C)-catalysed reaction in 1,4-dioxane [27]. Either non-activated esters, such as ethyl acetate, or symmetric or non-symmetric carbonates were used. High enantiomeric excesses and good yields were achieved with PSL-C and diallyl carbonate (DAC), whereas either no reaction occurred with different lipases or an equimolar diacylated product was formed with *Candida antarctica* type B (CAL-B). To this study followed a screening to assess the relationship between selectivity and the steric and electronic nature of the substituent in position 2 of propane diamines [28,29]. In particular, the authors underlined the importance of the aromaticity of the substituent for the enzymatic enantiorecognition mechanism. Prochiral pentane-1,5-diamines were also investigated as enzymatic substrates for PSL-C desymmetrisation and good results were obtained with ethyl methoxyacetate as the acyl donor in 1,4-dioxane [30].

Similar work, but with vicinal diamines, was carried out by Berkessel et al., where the stereoselective mono-Alloc (mono-Allyloxycarbonyl) protection of *cis*-1,2-diaminocyclohexane with lipase from CAL-B was reported [31]. Among the various solvents tried, toluene seemed to give the best result in terms of the reaction rate, although the authors reported 96 h of reaction time. *Meso*-1,2-diaryl-1,2-diaminoethanes were also assessed as enzymatic substrates with several lipases by Méndez-Sánchez et al [32]. Non-substituted diamine led to the highest conversion and enantiomeric excess of allyl aminocarbamate, while a moderate conversion was reached for *para*-substituted compounds using CAL-A and CAL-B. This result encouraged us to develop and optimise a biocatalytic procedure for the asymmetric mono-protection of *para*-chlorophenyl ethanediamine **5**, which is required for the preparation of Nutlin-3a (**1**).

The so-obtained desymmetrised diamine compound is an optically active core that can be found in a wide range of biologically active compounds besides Nutlin-3a [34]. This type of vicinal diamine is a versatile building block that can also be found in a plethora of important organic structures, such as organocatalysts, chiral auxiliaries and chelating agents [25,35,36,37]. Thus, a consistent biocatalytic methodology would permit the chemoenzymatic preparation of a broad library of derivatives, offering the possibility of orthogonal protecting strategies in mild and green operating conditions.

## 2. Results and Discussion

**Scheme 1:**
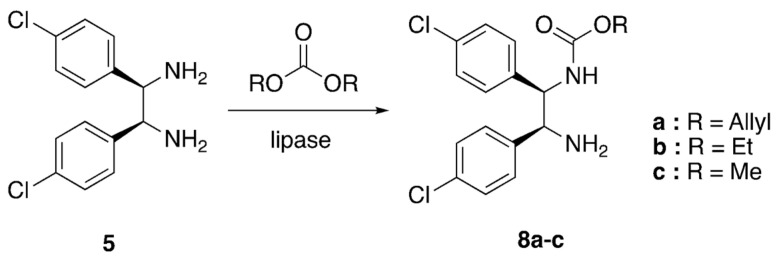
Lipase-catalysed asymmetric *meso*-diamine **5** desymmetrisation with green dialkyl carbonates.

With the aim of finding the most suitable conditions to perform the enzymatic reaction in Scheme 1 in terms of yields and enantiomeric excesses, we systematically tuned the reaction parameters, such as the lipase type, solvent, carbonate and temperature.

**Scheme 2:**
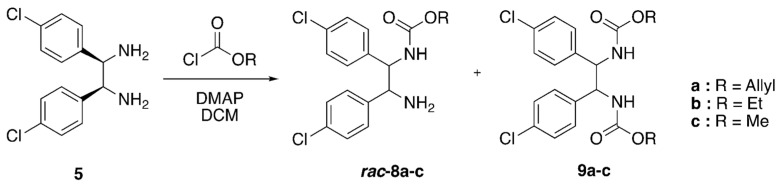
Racemic preparation of aminocarbamates with alkyl chloroformates with formation of undesired bis-carbamates.

Racemic products, which are required as reference compounds for the measurement of enantiomeric excess by chiral HPLC, were also synthesised. The non-enzymatic alkoxycarbonylation of prochiral *meso*-diamine was performed at room temperature with appropriate chloroformates in the presence of dimethylaminopyridine (DMAP). Under these reaction conditions, the favourite formation of the dicarbamate **9** as a byproduct dropped the yield of the isolated monocarbamate product. Therefore, in order to minimise dialkylation, the reaction mixture was cooled at 0 °C and kept at a low concentration, and the acylating agent was added portion-wise. Nevertheless, purification by flash chromatography was always necessary to separate the mixture of the mono- **rac-8** and the bisamide species **9** in a ratio of 4:1 (Scheme 2).

### 2.1. Enzyme Screening

In an attempt to select the best-performing enzyme for this reaction, several immobilised and free lipases were screened, including immobilised lipase from *Candida antarctica* type B, immobilised lipase from *Rhizomucor miehei* (RML), lipase from *Aspergillus niger* and *Penicillum roqueforti,* lipase from liver acetone powder porcine, immobilised lipase from *Pseudomonas cepacia* (PSL-C), immobilised lipase from *Candida rugosa* and Amano lipase from *Pseudomonas fluorescens*. Standard conditions were used in the initial activity test, namely, 60 mg of diamine **5**, 2 mL of DAC (concentration 30 mg/mL) and 200 mg of enzyme (amine/enzyme ratio 0.3:1) at 45 °C. Among these, *Candida antarctica* type *B* (CAL-B), *Rhizomucor miehei* (RML) and, in a lesser extent, liver acetone powder porcine were found to be able to catalyse the reaction. According to previously reported data, the best selectivity was achieved using CAL-B lipase immobilised on acrylic polymeric support (Table 1, entry 1). Good enantiopreference and no racemisation through migration of the carbamate group was observed on the Alloc derivative **8a**. Conversely, only product traces or no reaction were found with the other lipases under these reaction conditions. 

CAL-B is known to exhibit a more accessible binding site with respect to other lipases, as it displays a rather short lid in the closed form. This likely explains its higher activity towards a particular substrate, such as compound **5**.

The same preliminary screening protocol was also applied to diethyl carbonate (DEC) desymmetrisation. Similar to DAC desymmetrisation, the monocarbammate product **8b** was present in traces after three days using the enzyme from CAL-B and *Pseudomonas cepacia* (PSL-C), while other enzymes showed no significant activity towards diamine **5**. Likewise, in the reaction with dimethyl carbonate (DMC) as the acylating agent, traces of product **8c** were detected only when using lipase from *Pseudomonas fluorescens* (PSL-C) and CAL-B.

As the best-performing enzyme, CAL-B was investigated in the following experiments to further study the impact on its activity in our model reaction when changing the other parameters.

### 2.2. Effect of Solvent

Since the organic carbonates are in a liquid state at reaction temperatures, an organic solvent in this experiment would only act as a co-solvent, helping the solubilisation of *meso*-diamine and allowing for smooth stirring of the enzyme. Also, a good co-solvent is especially desired in scaled-up syntheses when large quantities of high-priced carbonate are needed. However, it is known that in some cases, solvents used as reaction media could generate the phenomena of enzymatic instability and deactivation. Gotor et al. screened a series of organic solvents in their system using PSL-C [30]. We carried out a similar study with our model to assess the effect of co-solvents on enzyme activity. We selected the most used organic solvents in enzymatic reactions that were able to efficiently solubilise our substrates. Diallyl carbonate and lipase from *Candida antarctica* B were chosen for this evaluation as an acyl agent and enzyme combination that led to the best conversion and enantiomeric excess. High-boiling solvents were preferred due to their suitability over a wide range of reaction temperatures (Table 2). It is important to note that after conducting a series of unsuccessful experiments with an initial diamine/enzyme ratio of 0.3:1, the enzyme loading was increased in this case until conversion was detectable. The minimum supported CAL-B quantity required to ensure reliable outcome was 1 g (0.06:1 diamine/enzyme ratio). Both toluene and 1,4-dioxane proved to be good solvents that allowed for a monophasic environment but resulted in not being well tolerated by the enzymes. The tertiary alcohol 2-methylbutan-2-ol (*tert*-amyl alcohol) was also tested as a reaction co-solvent. This low-toxicity solvent showed only a slight decrease in enzymatic activity, giving 23% as an isolated yield, making it a potential alternative for scale-ups in industrial application when expensive carbonates are needed.

With the purpose of studying the enzymatic reaction on a milligrams scale, we mainly worked solventless; thus, for the following reactions setup, the stoichiometric excess of carbonates not only acted as a reactant but also as a reaction solvent.

### 2.3. Effect of Temperature

Previous reported works conducted diamine desymmetrisation from room temperature up to 45 °C [27,28,29,30,31,32]. Hence, reference reactions in the present study were performed at 45 °C, as it also represents the optimal reaction temperature of most lipases. However, temperature is an important parameter that can affect enzymatic processes; thus, we tested the efficiency after we widened the temperature range. In particular, to gain new insights into the thermal adaptability and robustness of CAL-B and determine the most favourable conditions with substrate **5**, we opted to test its activity at a higher temperature (i.e., 75 °C), and slightly above 80 °C, which is its known upper operational limit.

Interestingly, we observed that supported enzymes, such as CAL-B, not only could tolerate high temperature but also led to substantial improvement in the conversion rate towards monocarbamates and enabled halving the reaction time without affecting the enantiomeric excess. Similar behaviour was observed using the three chemically diverse carbonates, DAC (Table 3, entries 1 and 3), DEC (Table 4, entries 1 and 2) and DMC (Table 5, entries 1 and 2). Specifically, keeping the reaction temperature at 75 °C led to moderate-to-good yields. This optimal operational temperature was maintained for the following experiments.

On the other hand *Pseudomonas cepacia*, *Candida rugosa* and non-supported *Pseudomonas fluorescens*, did not display the same trend, probably due to enzyme instability and irreversible denaturation at high temperatures.

### 2.4. Effect of Reaction Time

The mono-derivatisation of diamines is particularly challenging since the resulting monoalkylated compound is usually more reactive than the starting diamine, leading to an unwanted bis-alkylated compound in a short reaction time. Such bis-carbamate formation is rather fast in a non-enantioselective process, and, as mentioned above, compound **9** was observed as an insoluble precipitate when oxycarbonyl chlorides, such as methyl, ethyl and allyl chloroformates, were used as acylating agents.

Nonetheless, in accordance with the literature [31,32]. the enantioselective biocatalysed process is slower but yet more selective, yielding only the desired mono-alkoxycarbonylated product **8**. Conversely to the racemate preparation, we observed slow kinetics in the enzymatic reaction with carbonates, as it required several hours, but no bis-acylation occurred. However, the reaction time was significantly affected by the operational temperature, as we could accelerate the process by increasing the temperature to 75 °C.

### 2.5. Scaling Up

We assessed the possibility to scale up our method to ensure the process remained efficient and safe with a higher diamine concentration and lower catalyst loading. We observed no significant decrease in the yield and enantiomeric excess (Table 3, entries 1–2 and 4; Table 4, entries 3 and 4; Table 5, entries 2–5).

However, it is also important to note that the excess of carbonates can be easily recovered, both by distillation and after chromatographic purification. Similarly, CAL-B can potentially be reused after filtration since it is immobilised, and thus, particularly stable.

## 3. Materials and Methods

The biocatalysts *Candida antarctica* lipase type B (Novozyme 435^®^, CAL-B, 9000 PLU/g propyl laurate unit) and immobilised lipase from *Rhizmucor miehei* (275 IUN/g interesterification unit) were provided by Novozymes (Lyngby, Denmark). All other lipases, namely, Amano Lipase from *Pseudomonas fluorescens* (≥20,000 U/g), Lipase from *Pseudomonas cepacia* immobilised on immobead 150 (≥900 U/g), Lipase from *Candida rugosa* immobilised on immobead 150 (≥100 U/g) and Porcine liver acetone powder (50,000 U/g) (purchased from Sigma-Aldrich), as well as lipase from *Aspergillus niger* (194 U/g) and lipase from *Penicillium roqueforti* (650 U/g) (purchased from Biochemia Fluka) were used as received.

Thin-layer chromatography (TLC) for reaction monitoring was performed on precoated plates of silica gel Macherey-Nagel SIL Poligram/UV 254 (Merck, Darmstadt, Germany). For the TLC stain, 2% KMO_4_ aqueous solution was used.

Instruments: Mass spectra were recorded by an ESI single-quadrupole mass spectrometer Waters ZQ 2000 (Waters Instruments UK Ldt, 4 Tram Rd, Pontllanfraith, Blackwood, UK). Reaction conversions of non-isolated products were determined with a Backmann System Gold 168 HPLC apparatus with LC Column Kinetex 5 µm EVO C18 100 Å (250 Å, 4.6 mm) and a UV wavelength detector fixed at 220 nm.

The products were purified with a medium-pressure chromatography system Isolera 1 (Biotage, Sweden); the elution system and conditions used for each sample are reported in the experimental section.

^1^H (400 MHz) NMR and ^13^C (101 MHz) NMR spectra were obtained at an ambient temperature using a Varian 400 MHz spectrometer and chemical shifts δ, expressed as part per million (ppm), were referenced to residual ^1^H signals of the deuterated solvents (δ ^1^H 7.26 for CDCl_3_, δ ^1^H 2.50 for DMSO-*d*_6_ and δ ^1^H 3.31 for CD_3_OD). Peak assignments were aided by DEPT experiments. Coupling constants (*J*) are reported in hertz and the following abbreviations were used to describe the multiplicities and shapes of the peaks: s—singlet; d—doublet; dd—double doublet; t—triplet; td—triple doublet; m—multiplet.

Chiral separations for enantiomeric excess determination were performed under normal phase conditions on an Agilent 1100 Series; the chiral columns type and elution methods (i.e., normal phase composition, flow rate) used for each sample are specified in the experimental section.

The exact mass of the synthesised compounds was assessed by injecting 1 µL of 1 mg/mL of each sample into a Vanquish Flex Ultra High-Performance Liquid Chromatography (UHPLC) system coupled to a High-Resolution Orbitrap Exploris 240 mass spectrometer (ThermoFisher Scientific, Waltham, MA, USA). All samples were analysed in positive mode.

Enzymatic reactions were performed with the orbital shaker Incubator Innova^®^ 40 (New Brunswick, Canada).

The specific rotation values of optically active aminocarbamates were measured using a Polartronic H Schmidt + Haensch Polarimeter.

All characterisation data, comprising exact mass spectra, ^1^H (400 MHz) NMR and ^13^C (101 MHz) NMR spectra and chiral chromatographic separation are reported in Supporting Materials.

### 3.1. Enzyme Screening Protocol

A 30 mg/mL solution of *meso*-diamine **5** (60 mg, 0.2 mmol) in 2 mL (77.4 equiv.) of diallyl carbonate (DAC) was placed in an oven-dried Erlenmeyer flask under an inert air atmosphere and 200 mg of described lipase were weighted and added (diamine/enzyme ratio in weight 0.3:1). The mixture was gently shaken at 45 °C in an orbital shaker for six days at 250 r.p.m. The reaction was monitored by HPLC and mass spectrometry. When conversion was detected, the mixture was cooled to room temperature and the reaction was filtered to remove the enzyme and rinsed twice with DCM. After the solvent was evaporated, the yield of enantioenriched monocarbamate product **8a** was determined after silica gel chromatography purification (gradient solvent system: 30–100% Petroleum ether/AcOEt with 0.1% of NH_4_OH additive); otherwise, conversion was determined by HPLC peaks integration.

### 3.2. General Enzymatic Reaction

The enzymatic reactions were performed as described in the enzyme screening protocol and nominal quantities of *meso*-diamine **5**, CAL-B loading (expressed as diamine/enzyme ratio in weight), reaction time, temperatures, and solution concentration in DAC, DEC and DMC are reported in Table 3, Table 4 and Table 5, respectively.

### 3.3. General Racemic Reaction

A previously oven-dried 250 mL round bottom flask under Ar atmosphere was filled with a solution of *meso*-diamine **5** (500 mg, 1.78 mmol) and 240 mg (1.96 mmol, 1.1 equiv.) of DMAP in 10 mL of freshly distilled DCM. The mixture was cooled to 0 °C and a solution of alkyl chloroformate (1.25 mmol, 0.7 equiv.) in 20 mL of DCM was added dropwise through a dropping funnel over 30 min. The progress of the reaction was monitored both by thin-layer chromatography and mass spectrometry. When the limiting starting material was completely consumed, the solvent was evaporated and product **rac-8a-c** was purified from the crude material over silica gel in isocratic column chromatography (solvent system: AcOEt/Petroleum ether 1:1 with 0.1% of NH_4_OH additive).

*Allyl 2-amino-1,2,-bis(4-chlorophenyl)ethyl)carbamate* **8a**: TLC R_f_ (solvent system: AcOEt/Petroleum ether 1:1) = 0.28. ESI [M + H]^+^_calc_ = 365.0818, ESI [M + H]^+^_found_ = 365.0816, [M-NH_2_]^+^ = 348.06. FSC-HPLC Method A (Chiralpack-ID 250 mm × 4.6 mm, 5 µm. %MP (Hex:IPA) = 90:10. Flow = 1.5 mL/min. UV: 254 nm): t_R_[(1S,2R)-**8a**] = 11.43 min (major ent in enzymatic reaction), t_R_[(1R,2S)-**8a**] = 13.47 min (minor ent in enzymatic reaction). Method B (Whelk-01 SS 100 mm × 4.6 mm, 1.8 µm. %MP (Hex:IPA) = 80:20). Flow 1 mL/min): t_R_[(1S,2R)-**8a**] = 4.68 min (major ent in enzymatic reaction), t_R_[(1R,2S)-**8a**] = 12.00 min (minor ent in enzymatic reaction). ^1^H NMR (400 MHz, Methanol-*d*_4_) δ 7.40-7.25 (m, 8H), 5.77 (ddt, *J* = 16.2, 10.6, 5.4 Hz, 1H), 5.16–5.02 (m, 2H), 4.77 (d, *J* = 8.5 Hz, 1H), 4.36 (qdt, *J* = 13.5, 5.2, 1.6 Hz, 2H), 4.10 (d, *J* = 8.6 Hz, 1H). ^13^C NMR (101 MHz, CD_3_OD) δ 134.2, 130.5, 130.3, 129.7, 129.4, 117.3, 66.3, 62.1, 60.5. ${\left[\alpha \right]}_{D}^{\mathrm{20}}$ = + 12.5 (c 0.085, MeOH).

*Ethyl 2-amino-1,2,-bis(4-chlorophenyl)ethyl)carbamate* **8b**: TLR_f_ (solvent system: AcOEt/Petroleum ether 1:1) = 0.27. ESI [M + H]^+^_calc_ = 353.0818, ESI [M + H]^+^_found_ = 353.0817. FSC-HPLC: (Chiralpack-ID 250 mm × 4.6 mm, 5 µm. %MP (Hex:IPA) = 95:5. Flow = 1.5 mL/min. UV: 254 nm): t_R_[(1S,2R)-**8b**] = 6.24 min (major ent) in enzymatic reaction, t_R_[(1R,2S)-**8b**] = 9.12 min (minor ent in enzymatic reaction). ^1^H NMR (400 MHz, DMSO-*d*_6_) δ 7.62 (d, *J* = 9.3 Hz, 1H), 7.43–7.24 (m, 8H), 4.54 (t, *J* = 8.9 Hz, 1H), 3.98 (d, *J* = 8.4 Hz, 1H), 3.79 (p, *J* = 6.7 Hz, 2H), 1.02 (t, *J* = 7.1 Hz, 3H). ^13^C NMR (101 MHz, dmso) δ 219.5, 197.7, 194.3, 155.3, 143.2, 140.3, 131.5, 131.1, 129.7, 129.2, 127.8, 127.6, 60.6, 59.6, 58.8, 14.4. ${\left[\alpha \right]}_{D}^{\mathrm{20}}$ = + 14.9 (c 0.085, MeOH).

*Methyl 2-amino-1,2,-bis(4-chlorophenyl)ethyl)carbamate* **8c**: TLC R_f_ (solvent system: AcOEt/Petroleum ether 1:1) = 0.3. ESI [M + H]^+^_calc_ = 339.0662, ESI [M + H]^+^_found_ = 339.0660. FSC-HPLC Method A: (Chiralpack-ID 250 mm × 4.6 mm, 5 µm. %MP (Hex:IPA) = 95:5. Flowrate = 1.5 mL/min. UV: 254 nm): t_R_[(1S,2R)-**8c**] = 7.95 min, t_R_[(1R,2S)-**8c**] = 10.19 min. Method B: (Whelk-01 SS 100 × 4.6 mm, 1.8 µm. %MP (Hex:IPA) = 80:20. Flowrate = 1 mL/min. UV: 228 nm): t_R_[(1S,2R)-**8c**] = 4.88 min (major ent in enzymatic reaction), t_R_[(1R,2S)-**8c**] = 14.80 min (minor ent in enzymatic reaction). ^1^H NMR (400 MHz, Chloroform-*d*) δ 7.3–7.2 (m, 4H), 7.0 (m, 2H), 7.0–6.9 (m, 2H), 5.7 (d, *J* = 8.2 Hz, 1H), 4.8 (s, 1H), 4.2 (d, *J* = 5.3 Hz, 1H), 3.6 (s, 3H), 1.6–1.4 (m, 2H). ^13^C NMR (101 MHz, cdcl_3_) δ 156.4, 140.2, 136.8, 133.7, 133.6, 128.9, 128.7, 128.5, 128.4, 59.3, 52.4. ${\left[\alpha \right]}_{D}^{\mathrm{20}}$ = + 16.0 (c 0.085, MeOH).

## 4. Conclusions

Desymmetrisation of *meso*-4-chlorophenyl ethanediamine **5** represents an ingenious approach for the asymmetric synthesis of potent antineoplastic compound Nutlin-3a (**1**) since it leads to a key enantioenriched precursor. This type of reaction, which consists of the loss of an element of symmetry in prochiral molecules, is a convenient, as well as challenging, way used in organic synthesis to obtain enantiopure products. In previous work, we chemically performed this transformation using a chiral thiourea organocatalyst [22]. Recent advances in biochemistry showed that enzymes can be an ideal catalyst for this type of reaction, thanks to their great enantio- and regioselectivity, besides the given advantages in terms of sustainability and low environmental impact linked to their biodegradability and possible reusability. In particular, lipases are by far the most applied enzymes, even in industrial processes. However, even though there are a number of published works regarding the enzymatic desymmetrisation of prochiral diesters, diols and anhydrides in the literature [25,26], diamines have remained almost elusive, and even more so vicinal *meso*-diamines.

Here, we studied the asymmetric desymmetrisation of this type of compound through the biocatalytic alkoxycarbonylation to selectively obtain carbamates **8a-c** with diverse aliphatic carbonates as acyl donors.

This research presents a significant advancement in the field of enzymatic desymmetrisation of vicinal *meso*-diamine. The developed method exclusively produces the desired monocarbamate **8**, which effectively eliminates the formation of the unwanted bis-alkylated compounds commonly observed in chemical transformations. This achievement marks a substantial improvement over traditional methods.

Additionally, our study explored the use of a variety of carbonates’ chain lengths, namely, diallyl, diethyl and dimethyl carbonates, thereby broadening the scope and applicability of the enzymatic process.

Through a preliminary screening of a series of commercially available lipases, we selected the immobilised type B lipase from *Candida antarctica* (CAL-B), as it gave the best results in terms of the yield and enantiomeric excess thanks to its particularly favourable characteristics of stability and promiscuity.

CAL-B activity was assessed in the presence of a series of co-solvents, and we found that *tert*-amyl alcohol slightly reduced the enzymatic conversion under optimised conditions, whilst other solvents showed a dramatic decrease in the enzyme activity. However, the high solubility of the substrate **5** in each of the three dialkyl carbonate used not only simplified the reaction setup but also worked in the absence of solvents, as it acted as a reaction medium too. Moreover, the excess of the dialkyl carbonate could be recovered, which enhanced the sustainability and the economic viability of the process.

Through optimisation of the reaction conditions, particularly by increasing the temperature from 45 to 75 °C, we successfully halved the reaction time without compromising the conversion and enantiomeric excess, and thus, addressed a major limitation of the otherwise slow enzymatic reactions reported in the literature. This was possible since immobilised lipase from CAL-B also has a high resistance and displayed activity under this reaction condition.

Furthermore, we demonstrated the scalability of this method by conducting reactions at higher concentrations. Overall, satisfactory-to-good isolated yields and enantiomeric excess were obtained with carbonates DAC, DEC and DCM and no dramatic decrease was registered using a different reaction scale.

Overall, this research provides a robust environmentally friendly and efficient method for enzymatic desymmetrisation of compound **5** and might serve as a spark for the almost unexplored vicinal diamine as an enzymatic substrate, setting a new standard in this field.

## Figures and Tables

**Table 1:** Enantioselective desymmetrisation of 0.2 mmol of diamine **5** with 2 mL of DAC and 200 mg of enzyme at 45 °C under inert air condition for six days.

Entry	Lipase	Yield (%)	Ee (%) ^c^
1	CAL-B	27 ^a^	76
2	RML	16 ^a^	69
3	Liver acetone powder porcine	6 ^a^	9
4	*Pseudomonas cepacia* (PSL-C)	2 ^b^	n.d.
5	*Candida rugosa*	4 ^b^	n.d.
6	*Pseudomonas fluorescens*	8 ^b^	n.d.
7	*Aspergillus niger*	/	/
8	*Penicillum roqueforti*	/	/

^a^ Isolated yields were determined after purification of **8a** by silica gel chromatography. Traces indicate the mass spectroscopy detection of the product, yet in minimal amounts. ^b^ Conversion determined by HPLC peaks integration. ^c^ Enantiomeric excesses were determined by chiral stationary phase HPLC. (n.d.: not determined).

**Table 2:** Enantioselective desymmetrisation of 60 mg (0.2 mmol) of compound **5** with 1 mL of DAC in 30 mL of solvent with 1 g of supported *Candida antarctica* type B (0.06:1 ratio with diamine) at 45 °C under an inert air condition.

Entry	Solvent	Yield (%) ^a^
1	Toluene	Traces
2	1,4-dioxane	Traces
3	*tert*-amyl alcohol ^b^	23%
4	ACN	-

^a^ Isolated yields were determined after purification of **8a** by silica gel chromatography. Traces indicate the mass spectroscopy detection of the product, yet in minimal amounts. ^b^ A total of 3,5 mmol of compound **5** with 5 mL of DAC (100 mg/mL) at 75 °C.

**Table 3:** Enantioselective desymmetrisation of compound **5** with diallyl carbonate DAC in concentration range 30–70 mg/mL and supported CAL-B (in ratio with diamine) under inert air condition.

Entry	Diamine (mg)	Diamine/DAC (mg/mL)	Enzyme Loading ^a^	T (°C)	Time (Days)	Yield (%) ^b^ Ee (%) ^c^
1	60	30	0.3:1	45	6	27 76
2	200	50	0.5:1	45	6	14 83
3	60	30	0.3:1	75	3	65 80
4	500	70	0.5:1	75	3	65 89

^a^ Ratio of diamine/enzyme in weight. ^b^ Isolated yields were determined after purification of **8a** by silica gel chromatography. Traces indicate the mass spectroscopy detection of the product, yet in minimal amounts. ^c^ Enantiomeric excesses were determined by chiral stationary phase HPLC.

**Table 4:** Enantioselective desymmetrisation of compound **5** with diethyl carbonate DEC in concentration range 30–70 mg/mL and supported CAL-B (in ratio with diamine) under inert air condition.

Entry	Diamine (mg)	Diamine/DEC (mg/mL)	Enzyme Loading ^a^	T (°C)	Time (Days)	Yield (%) ^b^ Ee (%) ^c^
1	60	30	0.3:1	45	6	Trace n.d.
2	60	30	0.3:1	75	3	16 72
3	200	67	0.5:1	75	4	22 78
4	500	50	0.5:1	75	3	30 53

^a^ Ratio of diamine/enzyme in weight. ^b^ Isolated yields were determined after purification of **8b** by silica gel chromatography. Traces indicate the mass spectroscopy detection of the product, yet in minimal amounts. ^c^ Enantiomeric excesses were determined by chiral stationary phase HPLC. (n.d.: not determined).

**Table 5:** Enantioselective desymmetrisation of compound **5** with dimethyl carbonate DMC in concentration range of 30–70 mg/mL and supported CAL-B (in ratio with diamine) under inert air condition.

Entry	Diamine (mg)	Diamine/DMC (mg/mL)	Enzyme Loading ^a^	T (°C)	Time (Days)	Yield (%) ^b^ Ee (%) ^c^
1	60	30	0.3:1	45	6	trace n.d.
2	60	30	0.3:1	75	5	95 73
3	500	70	0.3:1	75	7	22 31
4	200	40	0.3:1	75	7	36 46
5	500	100	0.4:1	75	4	30 44

^a^ Ratio of diamine/enzyme in weight. ^b^ Isolated yields were determined after purification of **8c** by silica gel chromatography. Traces indicate the mass spectroscopy detection of the product, yet in minimal amounts. ^c^ Enantiomeric excesses were determined by chiral stationary phase HPLC. (n.d.: not determined).

## Data Availability

Data is contained within the article and Supplementary Material.

## Supplementary Materials

The following supporting information can be downloaded from https://www.mdpi.com/article/10.3390/molecules29143267/s1: Figure S1. ^1^H-NMR spectrum of compound **8a**; Figure S2. ^13^C-NMR spectrum of compound **8a**; Figure S3. Exact mass of compound **8a**; Figure S4. ^1^H-NMR spectrum of compound **8b**; Figure S5. ^13^C-NMR spectrum of compound **8b**; Figure S6. Exact mass of compound **8b**; Figure S7. ^1^H-NMR spectrum of compound **8c**; Figure S8. ^13^C-NMR spectrum of compound **8c**; Figure S9. Exact mass of compound **8c**; Figure S10. Chiral HPLC of **rac-8a**; Figure S11. Chiral HPLC of **8a**, Table 3, entry 1; Figure S12. Chiral HPLC of **8a**, Table 3, entry 2; Figure S13. Chiral HPLC of **8a**, Table 3, entry 3; Figure S14. Chiral HPLC of **rac-8b**; Figure S15. Chiral HPLC of **8b**, Table 4, entry 2; Figure S16. Chiral HPLC of **8b**, Table 4, entry 3; Figure S17. Chiral HPLC of **rac-8c**; Figure S18. Chiral HPLC of **rac-8c**; Figure S19. Chiral HPLC of **8c**, Table 5, entry 2; Figure S20. Chiral HPLC of **8c**, Table 5, entry 3; Figure S21. Chiral HPLC of **8c**, Table 5, entry 4; Figure S22. Chiral HPLC of **8c**, Table 5, entry 5.
