# Supplementary material for: Enzymatic Desymmetrisation of Prochiral meso-1,2-Disubstituted-1,2-Diaminoethane for the Synthesis of Key Enantioenriched (−)-Nutlin-3 Precursor
Source: Molecules. 2024 Jul 10;29(14):3267. doi: 10.3390/molecules29143267 (PMC11279714; doi:10.3390/molecules29143267)
Supplement: Supplementary file 1 [file molecules-29-03267-s001.zip › molecules-3081458-supplementary.pdf]

SUPPORTING INFORMATION

Enzymatic desymmetrisation of prochiral meso-1,2-disubstituted-1,2-diaminoethane for the synthesis of key enantioenriched (-)-Nutlin-3 precursor

Virginia Cristofori[1], Davide Illuminati[2], Chiara Bisquoli[1], Martina Catani[1], Greta Compagnin[1] Claudio Trapella[1*] and Anna Fantinati[3].

[1] Department of Chemical, Pharmaceutical and Agricultural Sciences, University of Ferrara, Via Luigi Borsari,46, 44121 Ferrara Italy. virginia.cristofori@unife.it; chiara.bisquoli@unife.it, trap@unife.it; Martina.catani@unife.it; greta.compagnin@unife.it.

[2] Department of Life Sciences, University of Modena and Reggio Emilia, Via G. Campi 213/d, 41125 Modena, Italy; davide.illuminati@unife.it

[3] Department of Environmental and Prevention Sciences, University of Ferrara, Corso Ercole I d'Este, 32 44121 Ferrara Italy. anna.fantinati@unife.it.

* Correspondence: Claudio Trapella, trap@unife.it; +39-0532-455924.

Table of contents:

Figure S1	^{1}H-NMR spectra of compound **6a**	**Pag.2**
Figure S2	^{13}C-NMR spectra of compound **6a**	**Pag.2**
Figure S3	Exact mass of compound **6a**	**Pag.3**
Figure S4	^{1}H-NMR spectra of compound **6b**	**Pag.4**
Figure S5	^{13}C-NMR spectra of compound **6b**	**Pag.4**
Figure S6	Exact mass of compound **6b**	**Pag.5**
Figure S7	^{1}H-NMR spectra of compound **6c**	**Pag.6**
Figure S8	^{13}C-NMR spectra of compound **6c**	**Pag.6**
Figure S9	Exact mass of compound **6c**	**Pag.7**
Figure S10	Chiral HPLC rac-**6a**	**Pag.8**
Figure S11	Chiral HPLC table 3 entry 1 **6a**	**Pag.8**
Figure S12	Chiral HPLC table 3 entry 2 **6a**	**Pag.9**
Figure S13	Chiral HPLC table 3 entry 3 **6a**	**Pag.9**
Figure S14	Chiral HPLC rac-**6b**	**Pag.10**
Figure S15	Chiral HPLC table 4 entry 2 **6b**	**Pag.10**
Figure S16	Chiral HPLC table 4 entry 3 **6b**	**Pag.11**
Figure S17	Chiral HPLC rac-**6c**	**Pag.11**
Figure S18	Chiral HPLC rac-**6c**	**Pag.12**
Figure S19	Chiral HPLC table 5 entry 2 **6c**	**Pag.12**
Figure S20	Chiral HPLC table 5 entry 3 **6c**	**Pag.13**
Figure S21	Chiral HPLC table 5 entry 4 **6c**	**Pag.13**
Figure S22	Chiral HPLC table 5 entry 5 **6c**	**Pag.14**

^{1}H-NMR spectra of compound **6a**

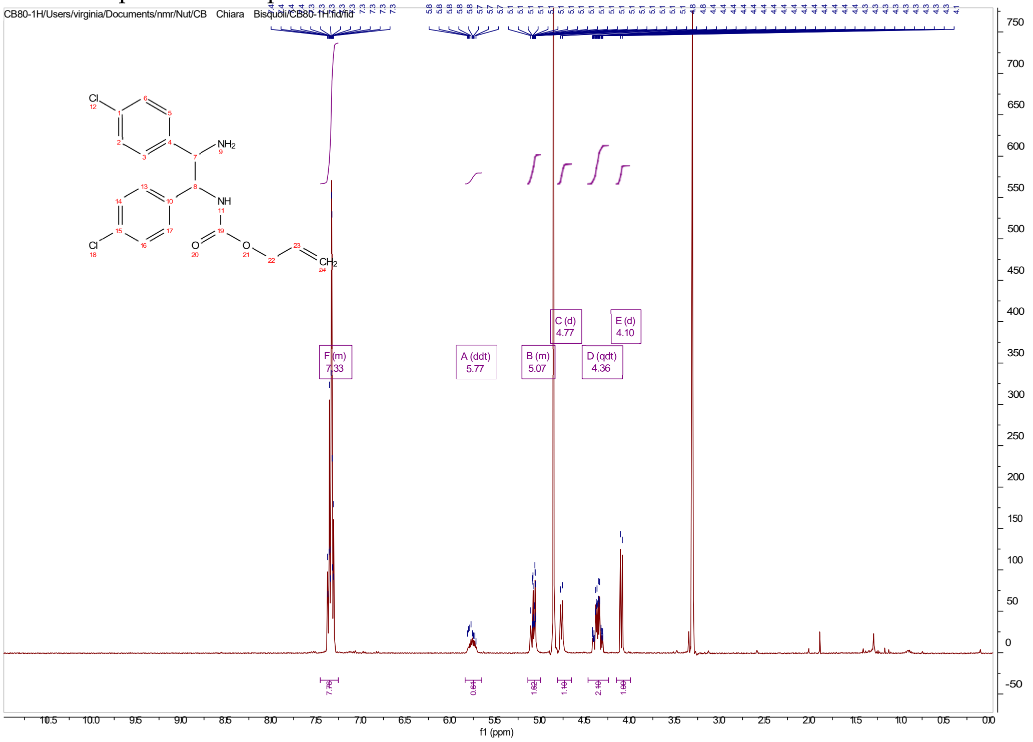

Figure S1. ^{1}H-NMR spectra of compound **6a.**

^{13}C-NMR spectra of compound **6a**

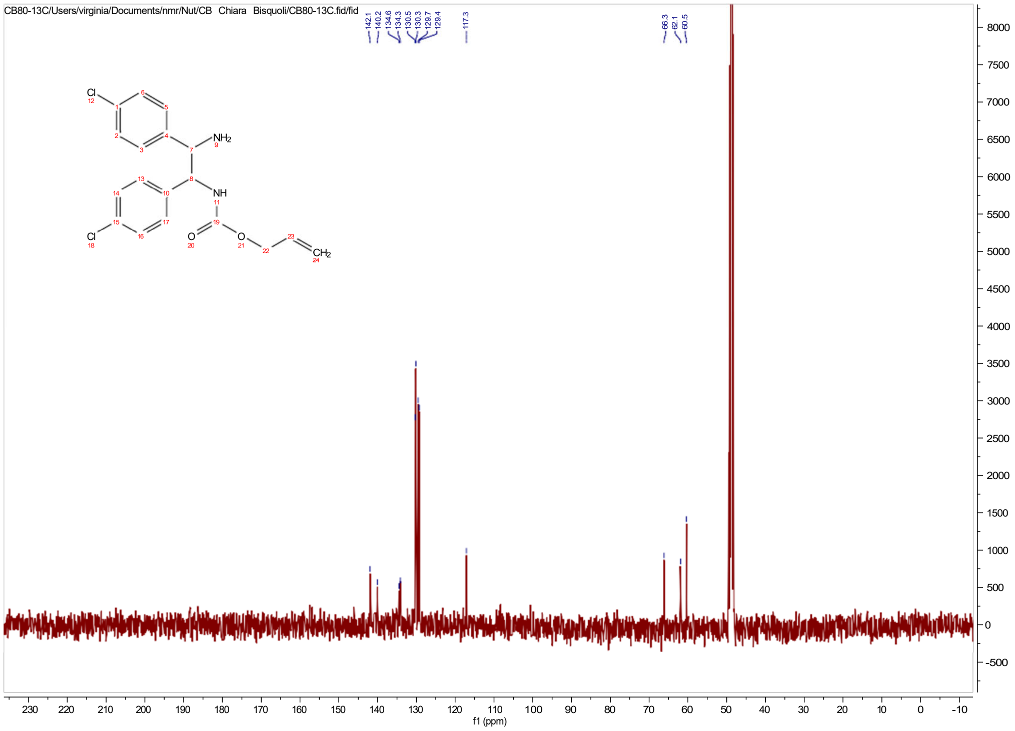

Figure S2. ^{13}C-NMR spectra of compound **6a.**

Exact mass of compound **6a**

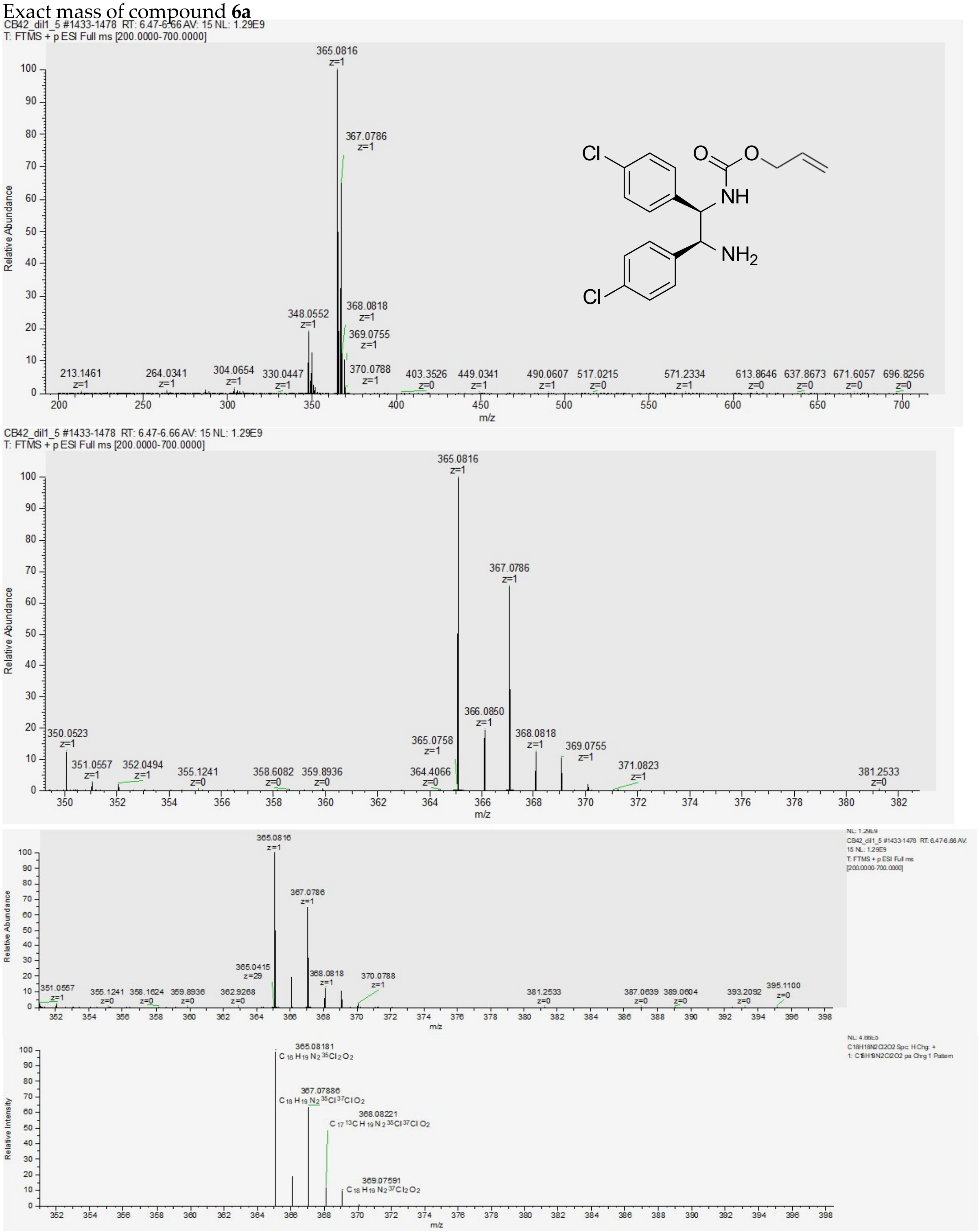

Figure S3. Exact mass of compound **6a**

^{1}H-NMR spectra of compound **6b**

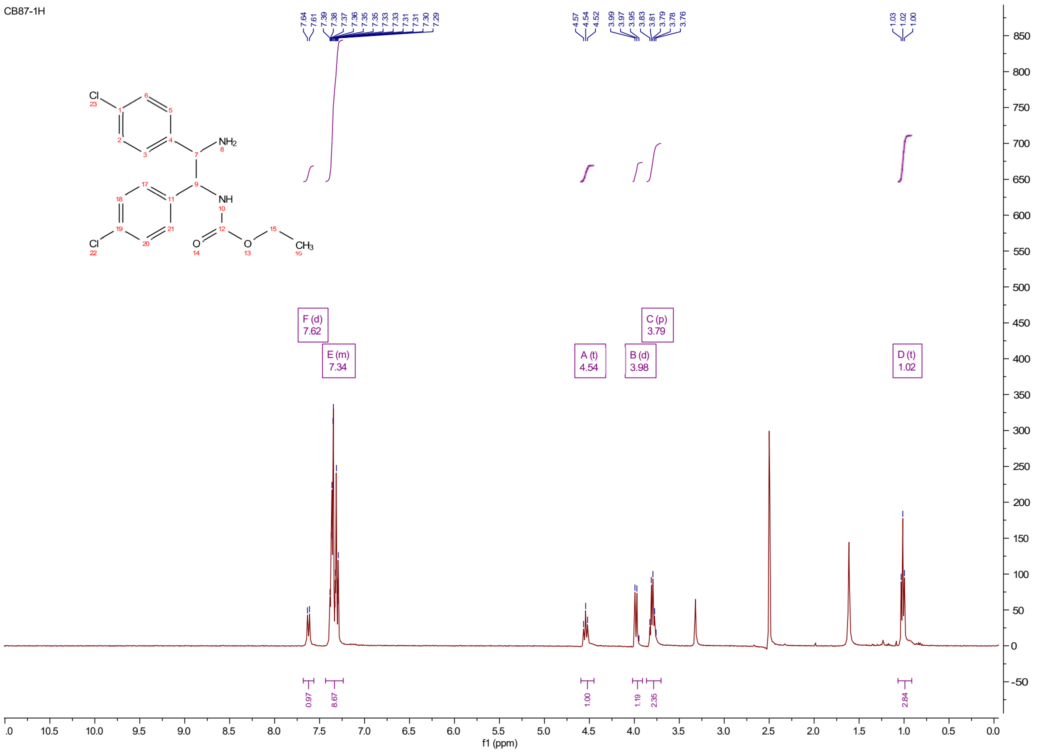

Figure S4. ^{1}H-NMR spectra of compound **6b**

^{13}C-NMR spectra of compound **6b**

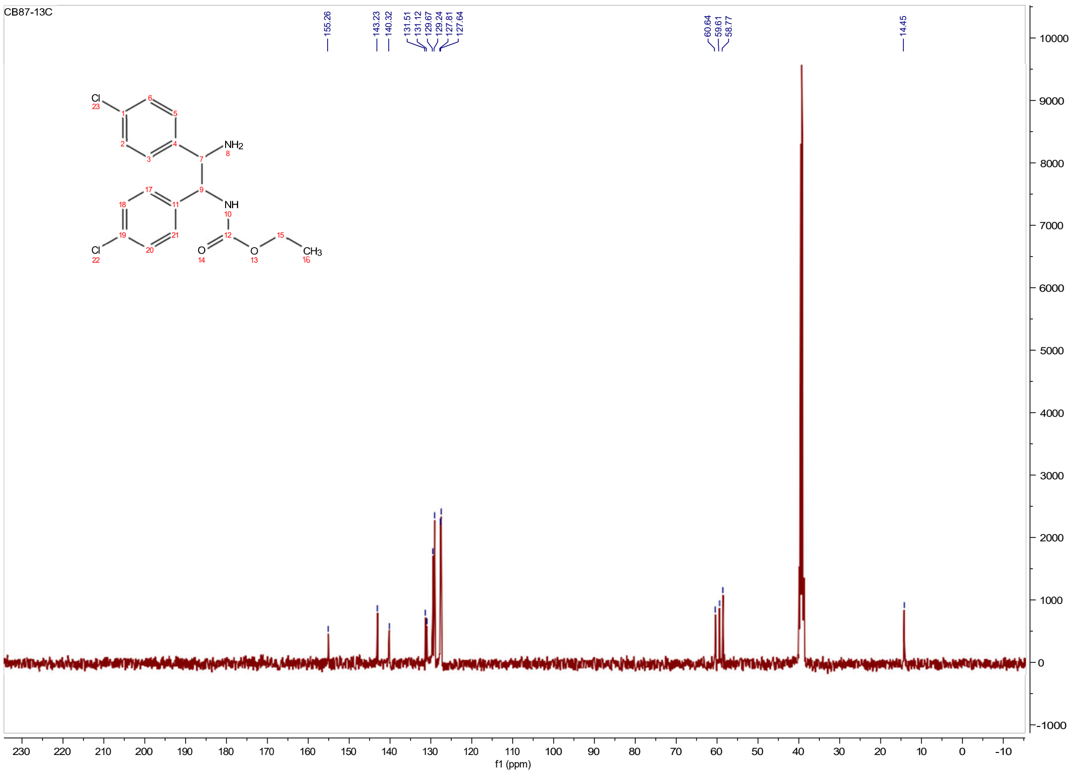

Figure S5. Exact mass of compound **6b**

Exact mass of compound **6b**

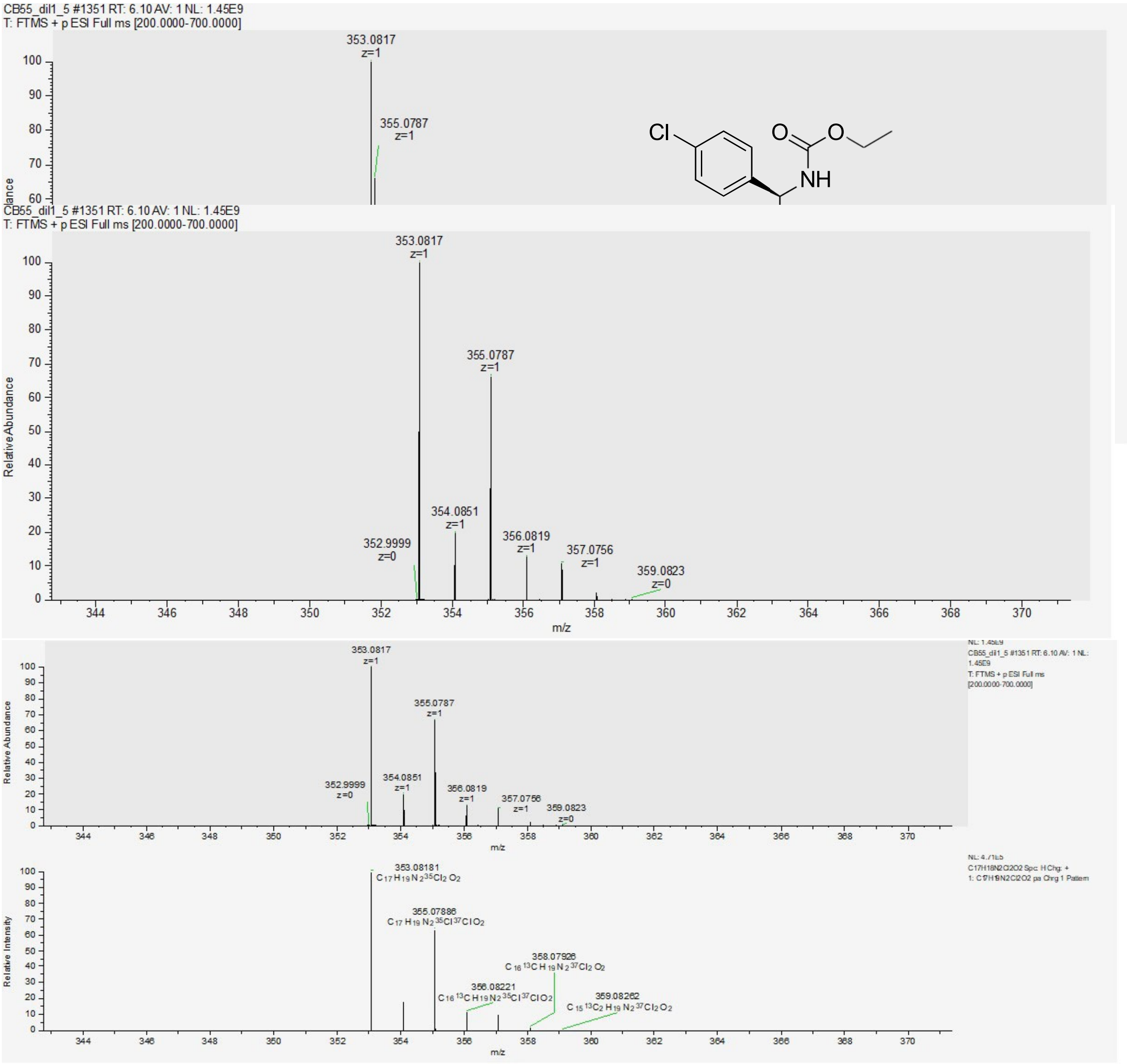

Figure S6. Exact mass of compound **6b.**

^{1}H-NMR spectra of compound **6c**

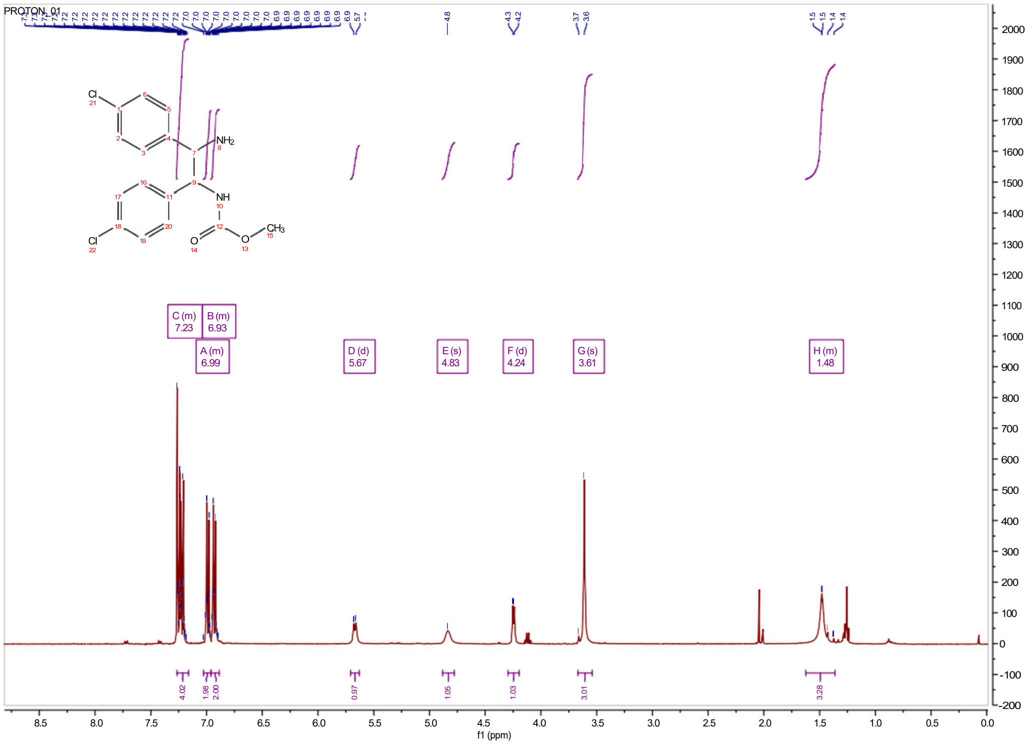

Figure S7. ^{1}H-NMR spectra of compound **6c.**

^{13}C-NMR spectra of compound **6c**

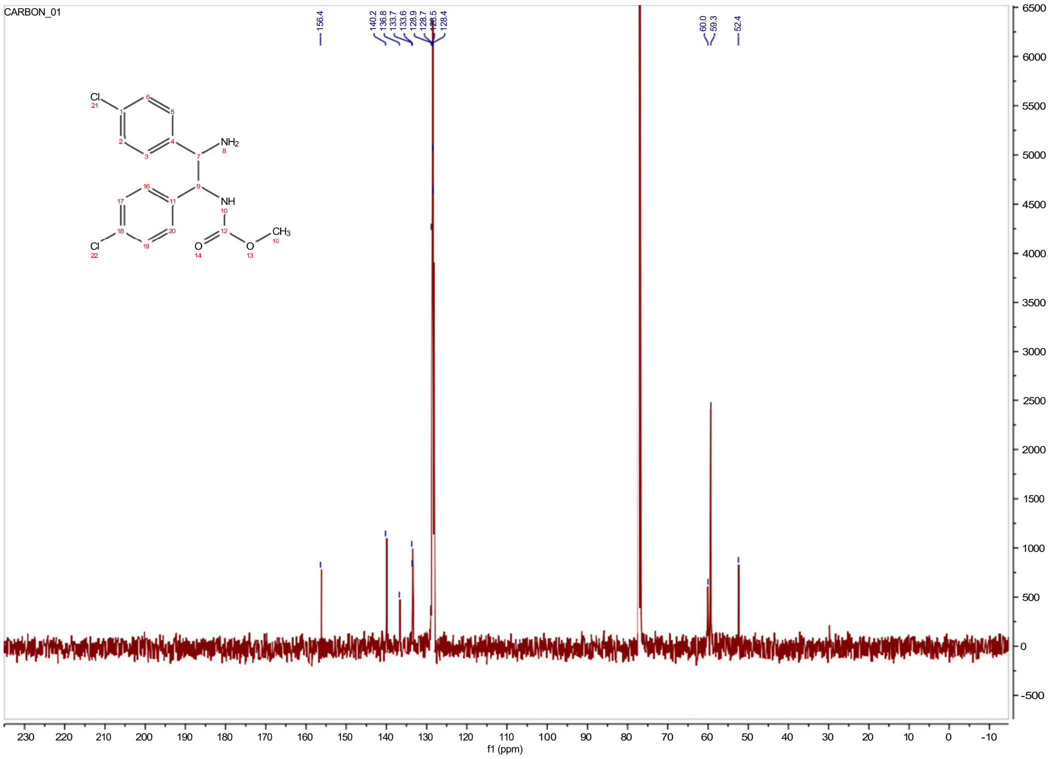

Figure S8. 13C-NMR spectra of compound **6c.**

Exact mass of compound **6c**

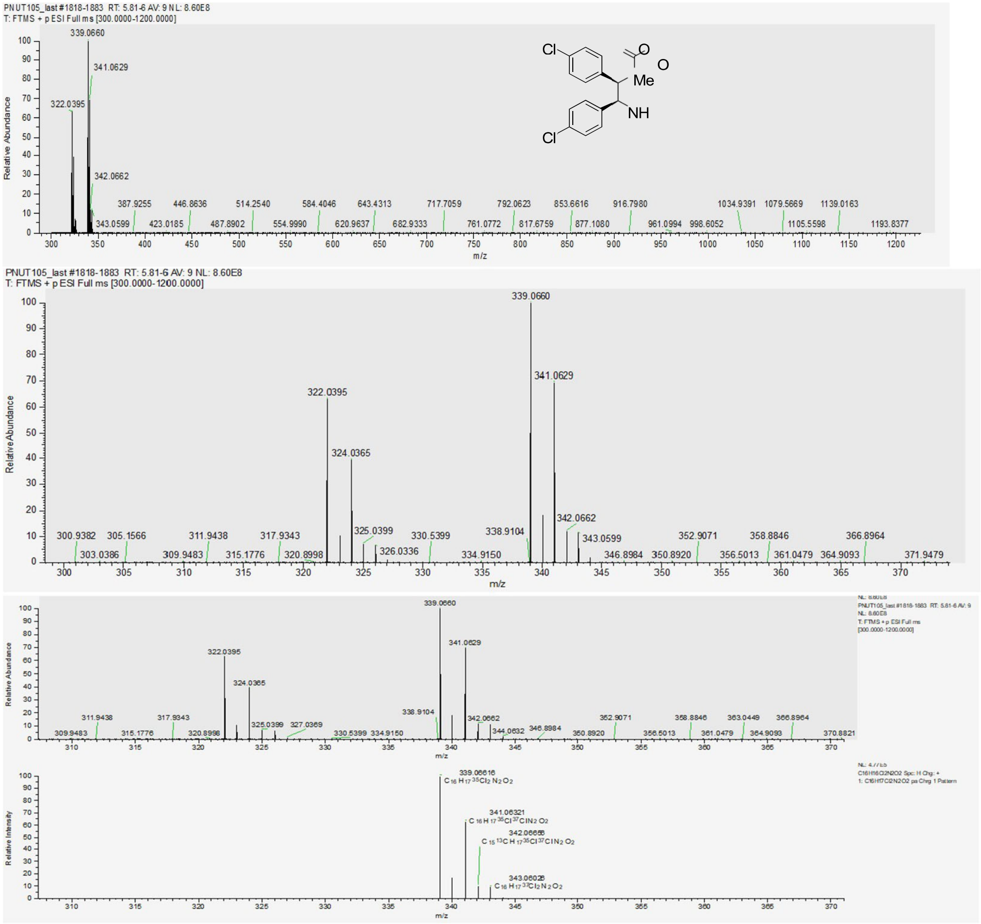

Figure S9. Exact mass of compound **6c.**

Compound	Calculated m/z	Theoretical m/z [M+H]$^{+}$	Δ (ppm)
**6a**	365.0816	365.0818	0.55
**6b**	353.0817	353.0818	0.28
**6c**	339.0660	339.0662	0.59

Chiral HPLC rac-**6a**

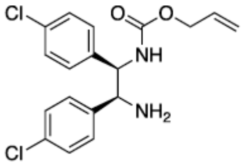

Whelk01 S,S
flow = 1ml/min
Hex : IPA = 80:20
A1= 13075,5   A2 = 12384,37   ee% = 2,7%

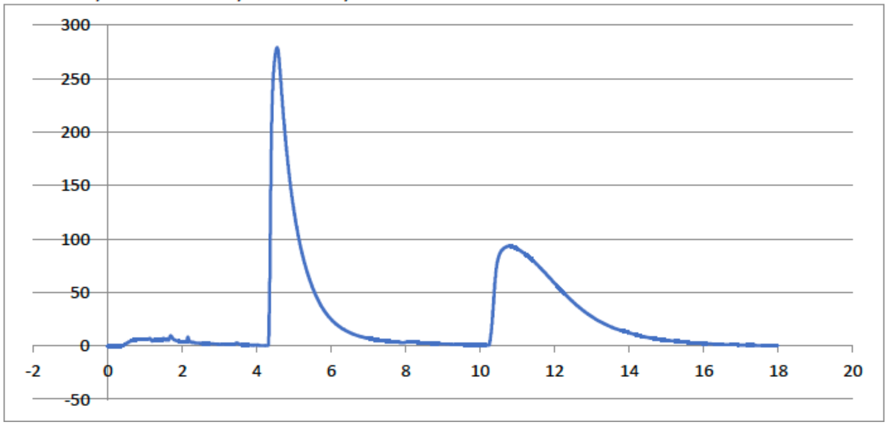

Figure S10. Chiral HPLC rac-**6a.**

Chiral HPLC Table 3 entry 1 compound 6a

Colonna: Chiralpak ID
Fv= 1,5 mL/min
%MP = 90:10
Area1= 31607,6   Area2= 4226
RT1=10,47 min   RT2=12,26 min
ee% = 76%

CAL-B 45°C 6gg

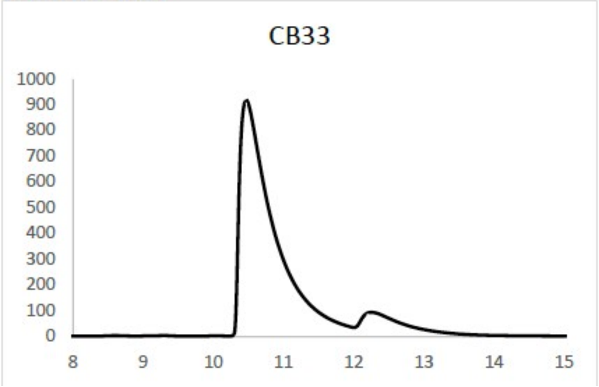

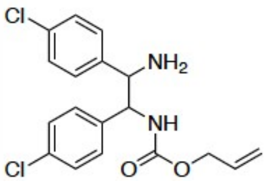

Figure S11. Chiral HPLC table 3 entry 1 **6a.**

Chiral HPLC Table 3 entry 2

Colonna: Chiralpak ID
Fv= 1,5 mL/min
%MP = 90:10
Area1= 9510,12 Area2= 864,671
RT1=11,43 min RT2=13,47 min
ee% = 83%

CAL-B 45°C 6gg

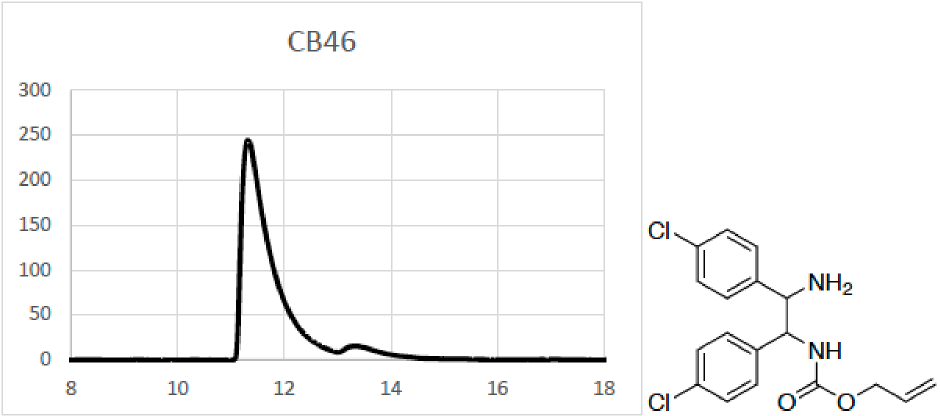

Figure S12. Chiral HPLC table 3 entry 2 **6a.**

Chiral HPLC Table 3 entry 3

Colonna: Chiralpak ID
Fv= 1,5 mL/min
%MP = 90:10
Area1= 3327,4 Area2= 354,862
RT1=11,84 min RT2=13,79 min

ee% = 80%

CAL-B 75°C 3 gg

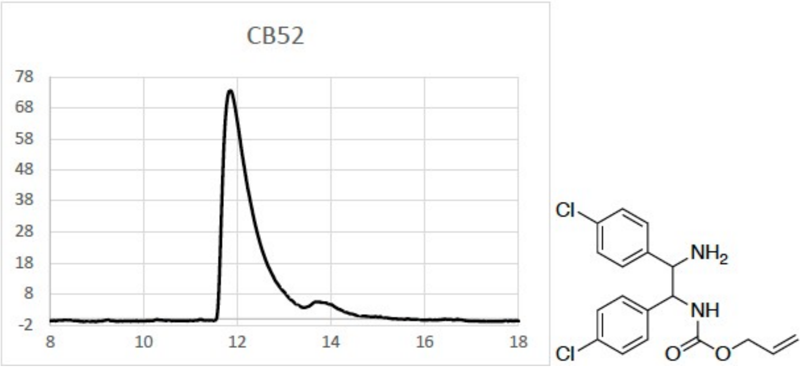

Figure S13. Chiral HPLC table 3 entry 3 **6a.**

Chiral HPLC rac-**6b**

COLONNA: Chiralpak ID
Fv = 1.5 mL/min
%MP = 95:5
Area1 = 9191.7 Area2 = 8949.2
Rt1 = 6.42 Rt2 = 8.45
ee% = 1.3%

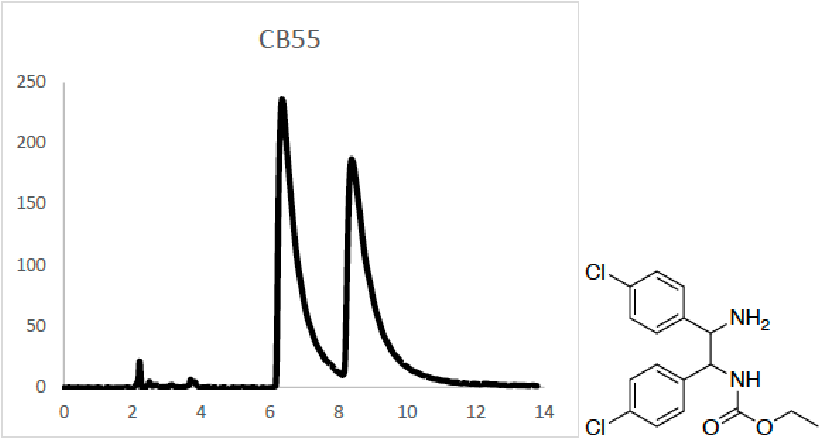

Figure S14. Chiral HPLC rac-**6b.**

Chiral HPLC Table 4 entry 2

COLONNA: Chiralpak ID
Fv = 1.5 mL/min
%MP = 95:5
Area1 = 5514.8 Area2 = 899.6
Rt1 = 6.68 min Rt2 = 9.52 min
ee% = 72%

CAL-B 75°C

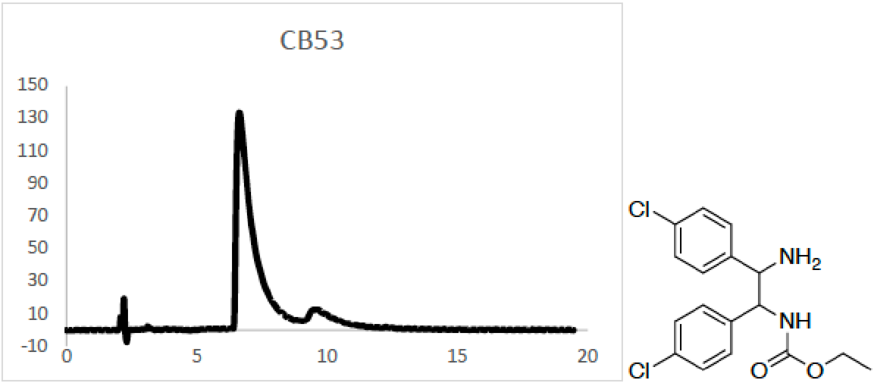

Figure S15. Chiral HPLC table 4 entry 2 **6b.**

Chiral HPLC Table 4 entry 3

COLONNA: Chiralpak ID
Fv = 1.5 mL/min
%MP = 95:5
Area1 = 16033.4 Area2 = 2010.5
Rt1 = 6.24 min Rt2 = 9.12 min
ee% = 77.7%

CAL-B 75°C

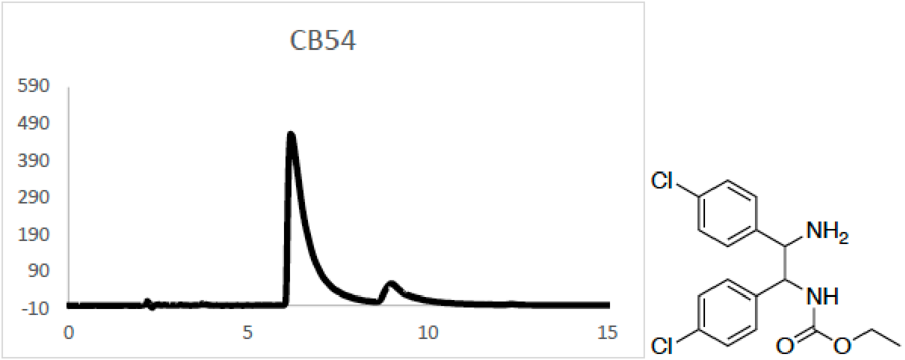

Figure S16. Chiral HPLC table 4 entry 3 **6b.**

Chiral HPLC rac-**6c**

COLONNA: Chiralpak ID
Fv = 1.5 mL/min
%MP = 95:5
Area1 = 6062.2 Area2 = 5975.6
Rt1 = 7.95 Rt2 = 10.19
ee% = 0.7%

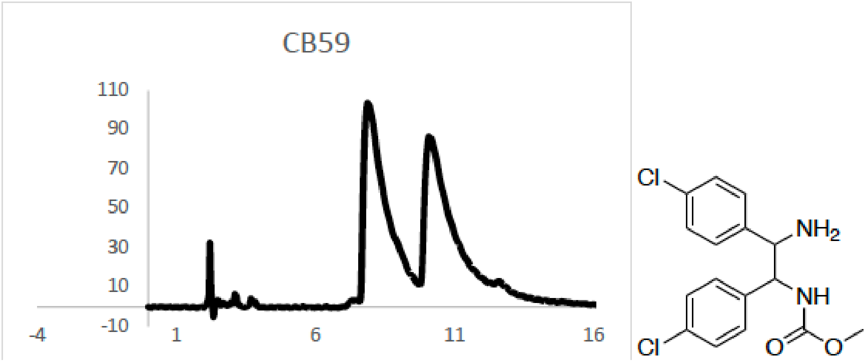

Figure S17. Chiral HPLC rac-**6c.**

Chiral HPLC rac 6c

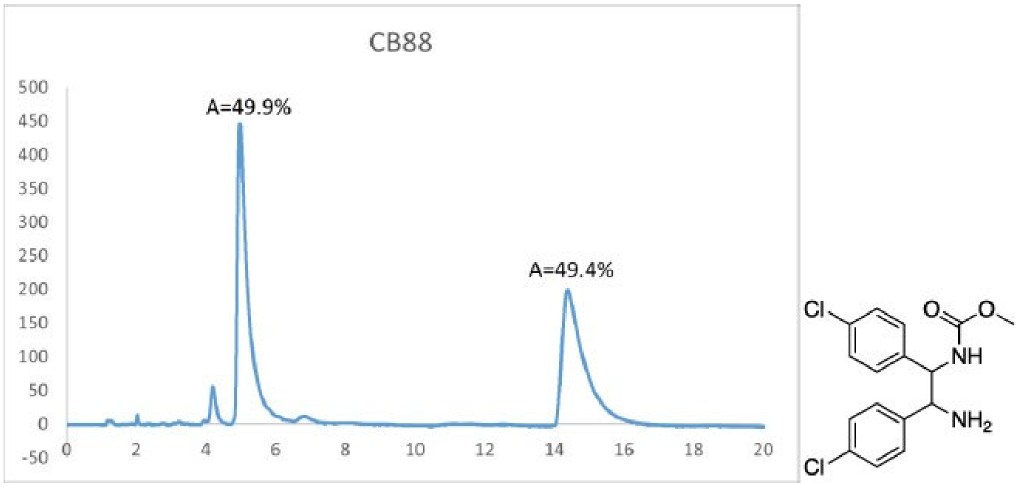

Figure S18. Chiral HPLC rac-**6c.**

Chiral HPLC 6c Table 5 Entry 2

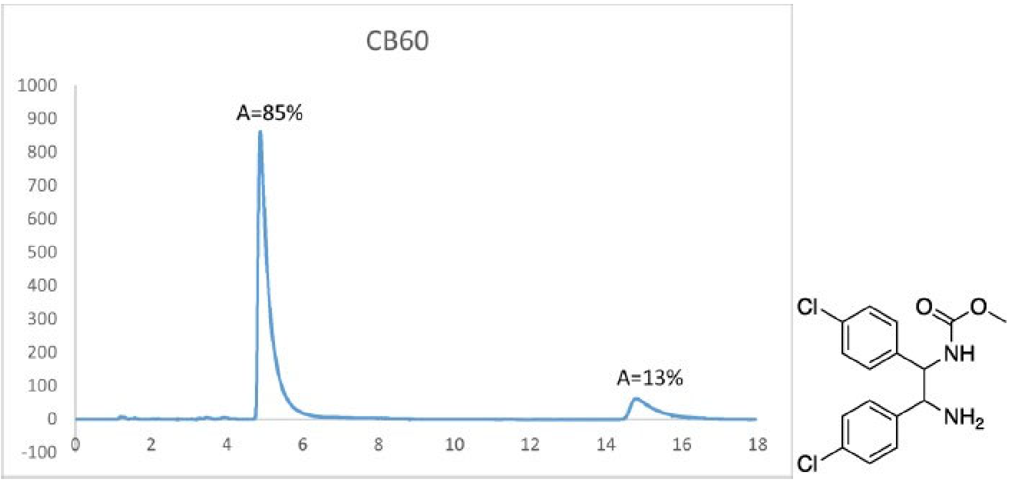

Figure S19. Chiral HPLC table 5 entry 2 **6c.**

Chiral HPLC 6c Table 5 Entry 3

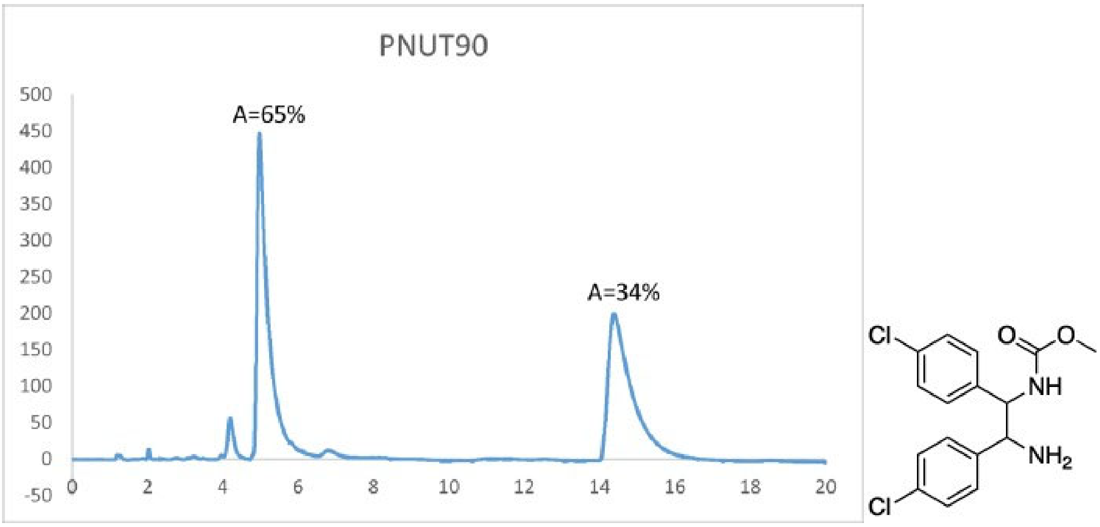

Figure S20. Chiral HPLC table 5 entry 3 **6c.**

Chiral HPLC 6c Table 5 Entry 4

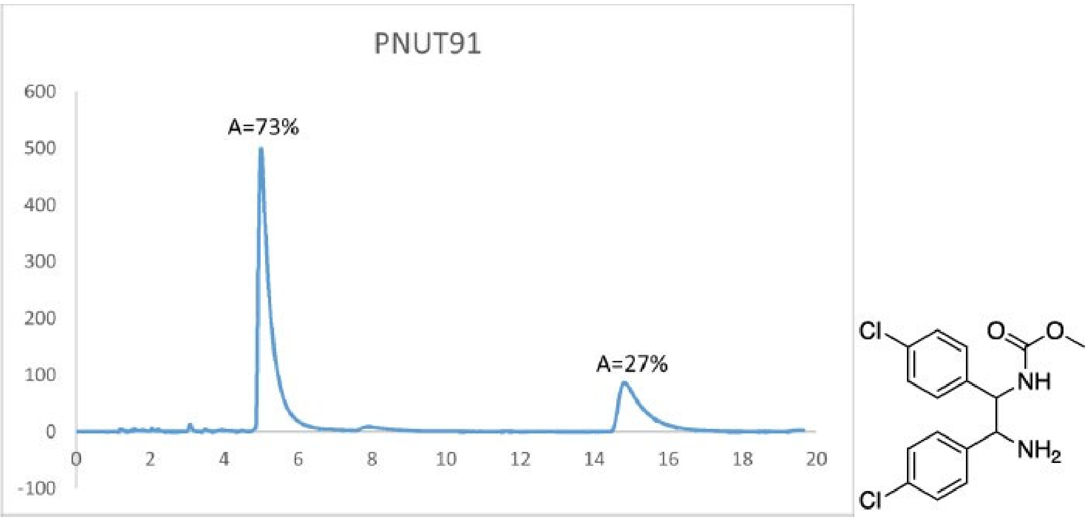

Figure S21. Chiral HPLC table 5 entry 4 **6c.**

Chiral HPLC 6c Table 5 Entry 5

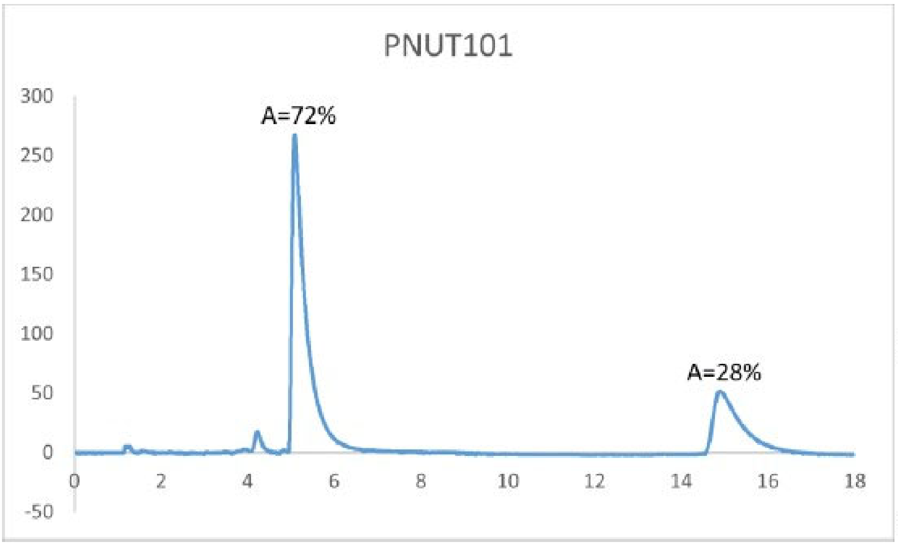

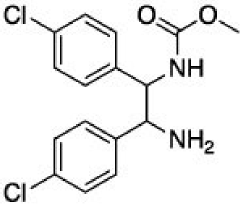

Figure S22. Chiral HPLC table 5 entry 5 **6c.**
